# Geopolymer Concrete with Lightweight Fine Aggregate: Material Performance and Structural Application

**DOI:** 10.3390/polym15010171

**Published:** 2022-12-29

**Authors:** Osama Youssf, Julie E. Mills, Mohamed Elchalakani, Fayez Alanazi, Ahmed M. Yosri

**Affiliations:** 1 Structural Engineering Department, Faculty of Engineering, Mansoura University, Mansoura 35516, Egypt; 2 UniSA-STEM, University of South Australia, Mawson Lakes Campus, Adelaide, SA 5095, Australia; 3 School of Civil, Environmental and Mining Engineering, The University of Western Australia, Perth, WA 6009, Australia; 4 Department of Civil Engineering, College of Engineering, Jouf University, Sakakah 72388, Saudi Arabia; 5 Civil Engineering Department, Faculty of Engineering, Delta University for Science and Technology, Belkas 11152, Egypt

**Keywords:** lightweight geopolymer concrete, rubber, vermiculite, LECA, reinforced slab bending

## Abstract

Limited information and data are available on the material and structural performance of GC incorporating lightweight fine aggregate. In this research, three types of lightweight fine materials were utilized to partially replace sand volume of GC. These lightweight materials were rubber, vermiculite, or lightweight expanded clay aggregate (LECA) and they were used in contents of 20%, 40%, 60%, and 100%. The variables were applied to better investigate the efficiency of each lightweight material in GC and to recommend GC mixes for structural applications. The concrete workability, compressive strength, indirect tensile strength, freezing and thawing performance, and impact resistance were measured in this study. In addition, three reinforced concrete slabs were made from selected mixes with similar compressive strength of 32 MPa and then tested under a 4-point bending loading regime. The results showed that using LECA as sand replacement in GC increased its compressive strength at all ages and all replacement ratios. Compared with the control GC mix, using 60% LECA increased the compressive strength by up to 44%, 39%, and 27%, respectively at 3, 7, and 28 days. The slabs test showed that partial or full replacement of GC sand adversely affected the shear resistance of concrete and caused premature failure of slabs. The slab strength and deflection capacities decreased by 9% and 30%, respectively when using rubber, and by 23% and 59%, respectively when using LECA, compared with control GC slab. The results indicated the applicability of GC mix with 60% LECA in structures subjected to axial loads. However, rubber would be the best lightweight material to recommend for resisting impact and flexural loads.

## 1. Introduction

The use of Portland cement concrete has been questioned in recent decades. This is due to the high rate of fossil fuel and limestone (natural resources) consumption, in addition to the high emission of carbon dioxide when Portland cement is produced [1,2,3]. The current production of Portland cement is responsible for 5–7% of the global carbon dioxide emission [4] and the production is expected to increase to 4.4 billion tons by 2050 [1]. 

The development of high-rise concrete buildings and the associated infrastructure, increase the concrete demand day after day, which raises concerns about the future sustainable use of Portland cement concrete [5]. Geopolymer concrete (GC) is an environmentally friendly alternative to traditional concrete. GC uses no cement, but uses silica-alumina rich materials instead, which are industry by-product materials. This significantly reduces the demand of cement and the associated carbon footprint [6,7,8,9,10,11].

Lightweight concrete is an economical type of concrete that can benefit structures through higher strength to weight ratio, lower cost of foundation, and improved resistance to fire [12,13,14,15,16]. Producing lightweight GC has the potential of reducing structural mass and hence the consumed concrete amount that can result in more sustainable construction. To date, limited research has been carried out to investigate the performance of lightweight GC. Coppola et al. [17] incorporated air-entraining admixture with lightweight glass in geopolymer mortar. The results showed that the manufactured mortar was able to reach 8 MPa compressive strength at 28 days. Aslani et al. [1] used fine rubber at 10% and 20% content, and expanded polystyrene at 25%, 50%, 75%, and 100% content to replace GC fine aggregate by volume. The GC compressive strength decreased with increasing polystyrene content, such that the strength dropped by 81% at a polystyrene content of 100%. The strength decreased by 37% when rubber was used at 20% content. A strength reduction of 60% was reported when combining 20% rubber and 25% polystyrene. Posi et al. [18] investigated the properties of GC containing recycled aggregate from lightweight blocks as a full replacement of mineral aggregate. This produced lightweight GC with density of 860-1400 kg/m^3^, but with limited compressive strength of about 16 MPa. The highest compressive strength was found when using fine lightweight aggregate of 2.3 times that of the coarse lightweight aggregate. Bhogayata et al. [19] replaced 25–100% of the total GC mineral aggregate by lightweight expanded clay aggregate (LECA) and reported no significant effect on compressive strength, tensile strength, or impact resistance when replacing mineral aggregates by up to 50%. 

Nano materials have also been shown to enhance the GC performance [20]. Raza et al. [21] used varying amounts of nano titanium dioxide into micro carbon fiber reinforced geopolymer pastes. They showed that the compressive and flexural strengths were improved by 23% and 40%, respectively. Alvee et al. [22] explored the influence of nano-silica and crystalline admixture (CAs) on the mechanical properties of geopolymer pastes. Their results demonstrated that adding nano-silica and CAs by 5% increased the compressive strength by 33.63%, flexural strength and fracture toughness by 232.69%. Alomayri [23] tested the mechanical properties of micro steel fiber reinforced geopolymer composites containing different amounts of nano-silica. Their results showed that the addition of 2% nano-silica significantly enhanced the mechanical properties of the composite.

Few studies have been carried out on the structural application of GC in reinforced slabs. Meng et al. [24] tested GC slabs under blast loads. All slabs were reinforced by steel rebars and some of them were additionally reinforced by steel fibres. The results showed that all slabs demonstrated an elastic structural response with visible cracks on slabs that had no steel fibers. However, slabs additionally reinforced by steel fibers showed much less visible cracks and good structural performance with a maximum central deflection of 1 mm. Meng et al. in another study [25] tested steel wire mesh reinforced GC slabs under blast loads. The results indicated that incorporating wire mesh reinforcement in GC enhanced the slab’s performance against blast loads. In addition, GC slabs experienced fewer cracks than those observed in the corresponding conventional concrete slabs. Mohana and Nagan [26] tested reinforced ferrocement GC slabs under flexural loads. It was concluded that the energy absorption, deformation at peak load, and load carrying capacity were higher when using ferrocement geopolymer instead of conventional ferrocement. 

As per the above literature, limited information and data are available on the effect of using lightweight fine aggregate as a partial replacement of GC sand on its performance at the material and structural levels. To address this research gap and to develop more sustainable concrete, eleven GC mixes were produced, tested, compared, and analyzed. The concrete mix properties, durability, and the structural behavior were investigated. The structural performances of reinforced GC slabs made of selected mixes and subjected to flexural load were also examined. The research aims to better investigate the efficiency of the lightweight aggregate in GC and to recommend GC mixes for structural applications.

## 2. Materials and Methods

### 2.1. Materials

**Figure 1:**
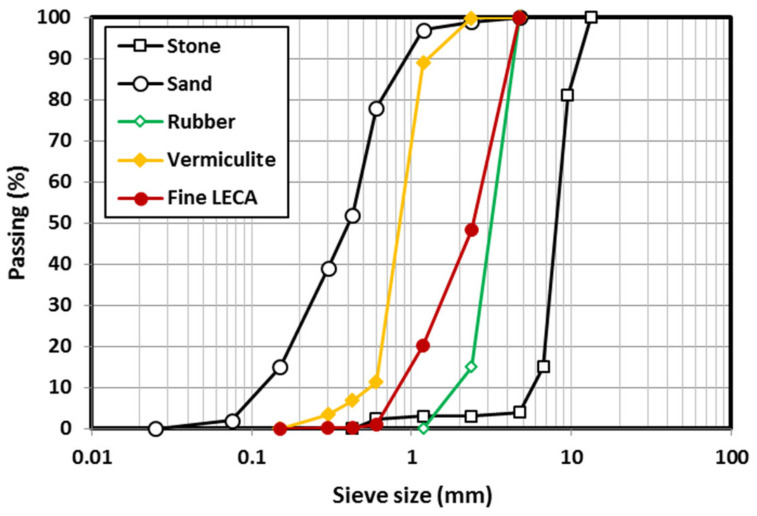
Particle size distribution for all aggregates used.

The binder materials used in all mixes were ground granulated blast furnace slag (GGBFS) and fly ash with chemical composition shown in Table 1. According to ASTM C618-15 [27], the summation of SiO_2_ + Al_2_O_3_ + Fe_2_O_3_ is more than 70% of fly ash and hence it is classified as Class F. The specific gravities of fly ash and slag were 2.57 and 2.83, respectively. The coarse and fine aggregates used in all mixes were 10 mm dolomite stone and river sand with maximum aggregate size of 4.75 mm, respectively. Three lightweight fine aggregates (rubber, vermiculite, or LECA) were used as partial replacement of GC sand. The aggregate particle sizes ranged between 2.36 mm and 4.75 mm for rubber, 0.3 mm and 2.36 mm for vermiculite, and 0.3 mm and 4.75 mm for LECA. Figure 1 shows the particle size distribution for all aggregates used in this study. The unit weight, specific gravity, and fineness modulus were 1590 kg/m^3^, 2.71, and 7.89, respectively for dolomite; 1420 kg/m^3^, 2.61, and 2.20, respectively for sand; 530 kg/m^3^, 0.97, and 4.85, respectively for rubber; 80 kg/m^3^, 0.24, and 2.97, respectively for vermiculite; and 880 kg/m^3^, 1.7, and 4.3, respectively for LECA. High range water reducer (superplasticizer—SP) and retarder with respective specific gravities of 1.085 and 1.057 were used in this study. A mix of sodium silicate (SS) and sodium hydroxide (SH) solutions was used as the activator in the GC mixes. The specific gravity of the alkaline solution was 1.28 and the mixing ratio of SS:SH was 1.7:1.0 by weight. The prepared 12 M sodium hydroxide solution was left to cool down for 2 h and then mixed with sodium silicate solution for 30 min using a mechanical liquid stirrer. The activator was left to cool down to room temperature for 24 h before use in the concrete mixing.

### 2.2. Mix Designs and Variables

GC mixes were designed with constant dolomite content of 611 kg/m^3^, constant activator content of 207 kg/m^3^, constant water content of 177 kg/m^3^, constant fly ash content of 206 kg/m^3^, constant slag content of 206 kg/m^3^, constant superplasticizer content of 9.4 kg/m^3^, and constant retarder content of 2.1 kg/m^3^. The sand content in the GC control mix was 611 kg/m^3^ and was partially replaced with rubber, vermiculite, or LECA in different mixes by volume. The fine to coarse aggregate ratio was 1:1.

**Figure 2:**
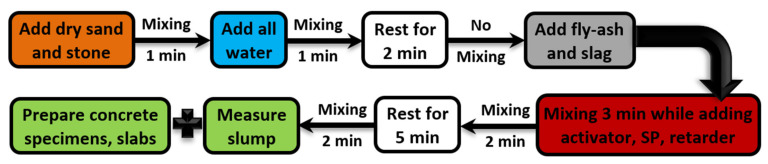
Geopolymer concrete mixing procedure.

The mixing procedure was as follows: mix dry sand and stone for 1 min.; add all water and mix for 1 min.; rest for 2 min.; add fly ash and slag and mix while adding the activator, superplasticizer, and retarder in 3 min and mix for additional 2 min. when all materials are in; rest for 5 min.; and mix for the final 2 min. It was observed that the GC appeared flowable after adding all materials and mixing for additional 2 min.; however, that flowability quickly decreased within 5 min. This was the reason for leaving the mixes for 5 min. to rest and then mixing them again before measuring the fresh concrete properties and filling the prepared moulds. Figure 2 summarizes the mixing procedure used in this experimental study.

The variables in this investigation were: lightweight fine material (rubber, vermiculite, LECA), and sand replacement ratio (20%, 40%, 60%, and 100%). The ingredients of concrete mixes in this study are shown in Table 2. Mix code “GC” stands for the control geopolymer concrete. The mix code for all other mixes starts with the sand replacement ratio followed by the lightweight material type; R, V, or L standing for rubber, vermiculite, or LECA, respectively.

### 2.3. Specimen Preparation and Test Procedures

Mechanical and durability characteristics were measured in this study including workability, unit weight, compressive strength (3, 7, and 28 days), indirect tensile strength, freezing-thawing influence, and impact resistance. From each mix, two 100 × 200 mm cylinders per measurement day were prepared to measure both the unit weight and the 3, 7, and 28 day compressive strength. Another two 100 × 200 mm cylinders were prepared to measure the 28 day indirect tensile strength. A standard hammer and rod were used in compacting the prepared concrete specimens. The concrete was left in the moulds for 24 hrs before de-moulding, labelling, and curing were performed according to AS1012.8.1 [28]. The ends of all cylindrical specimens tested in this study were ground according to AS 1012.9 [29].

**Figure 3:**
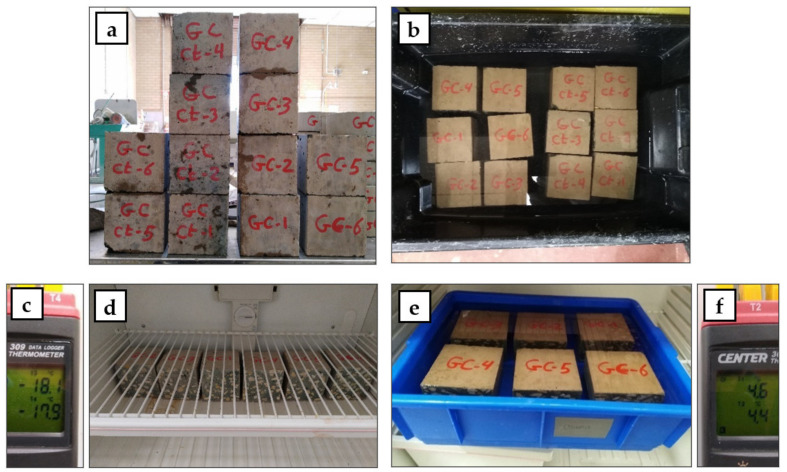
Freezing-thawing test specimens and procedures: (**a**) specimen preparation, (**b**) specimen curing, (**c**) freezing temperature, (**d**) specimen freezing, (**e**) specimen thawing, and (**f**) thawing temperature.

The freezing-thawing effect and the impact resistance were measured for mixes GC, 60 R, 60 V, and 60 L. The freezing-thawing effect on GC compressive strength was measured according to ASTM C666 (Procedure B) [30] using twelve 100 mm^3^ concrete cubes. The cubes were cut at 28 days concrete age from three 100 × 100 × 470 mm concrete prisms that were taken from each mix and kept in wet curing all the time until the test day. Half of the prepared cubes per mix were used for the freezing-thawing cycles and the other half were kept as control specimens. Three sets of freezing-thawing cycles (10, 20, and 30 cycles) were applied with a 4 hrs period each full cycle. For each freezing-thawing cycle, the specimens were picked up from the curing water and their surfaces were dried using a piece of cloth, then they were inserted in a freezer with a temperature of −18 °C for 2 h (freezing time). The specimens were then fully surrounded by water in a container and moved to a fridge with a temperature of 4 °C for 2 hrs (thawing time). At the end of each cycle set, the compressive strength was measured using two cubes and compared with another two control cubes that were left in clean tap water from after the 28 days until the end of each cycle set (test day). Figure 3 shows the details of the prepared freezing-thawing test specimens and procedures.

**Figure 4:**
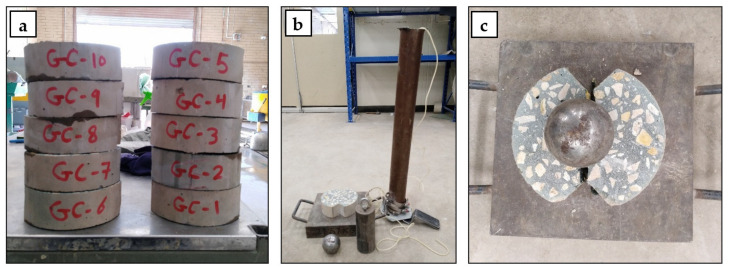
Impact resistance test details: (**a**) specimen preparation, (**b**) drop weight test tools, and (**c**) a tested specimen.

The performance of geopolymer concrete against impact load was measured according to ACI 544 [31] at 28 days concrete age. Ten discs from each mix were prepared with dimensions of 150 mm diameter, 50 mm thickness, and two 25 mm triangular notches, as can be seen in Figure 4. The impact resistance was calculated as the total number of blows needed to split the concrete disc into two halves.

**Figure 5:**
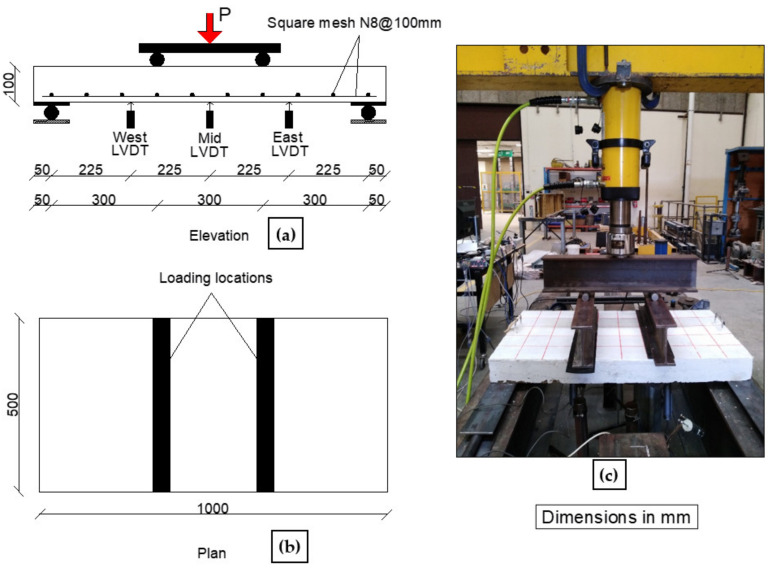
Slab geometry (**a**,**b**), reinforcement details (**a**), and test setup (**c**).

For the structural performance of GC slabs under 4-point bending, three steel reinforced slabs were cast from concrete mixes GC, 20R, and 100L. The selected mixes for the reinforced slabs had similar compressive strength of 32 MPa and were made to compare the structural behavior of the proposed lightweight GC mixes with that of the control GC mix. Figure 5 shows the slab geometry and reinforcement details designed according to AS 3600 [32]. Each slab specimen had a 100 mm thickness, 500 mm width, and 1000 mm length (900 mm clear span). Square mesh N8@100 mm spacing was the reinforcement of each slab. This corresponds to a 0.5% ratio of longitudinal reinforcement. The yield strength, yield strain, and modulus of elasticity for the N-grade reinforcement used were 550 MPa, 0.00275, and 200 GPa, respectively.

The reinforced slabs were cast in wooden moulds and left for 48 h before de-moulding and were then cured for 28 days in a water bath. White paint and grids were applied on the slabs to facilitate observation of cracks, see Figure 5. The slabs were tested under 4-point bending monotonic load using a hydraulic jack (600 kN capacity and 150 mm stroke) with 5 kN/min loading rate. The slabs were instrumented with 3 linear variable differential transformers (LVDT) to measure the deflection at several locations. One LVDT was located at the beam mid-span (450 mm from beam supports) and another two LVDTs were located at 225 mm from the beam supports (West and East LVDTs). The load was measured using a 500 kN loading cell. A data acquisition system (HBM Quantum-X MX1615, made in Germany) and LABVIEW 8.6 [33] were used in controlling and recording the measurements of the tested slabs.

## 3. Results and Discussion

In this research, eleven GC mixes were produced, tested, compared, and analyzed. In addition, the structural performance of GC slabs made of selected mixes was tested and compared. Concrete characteristics including fresh and hardened durability and mechanical properties were determined such as workability, compressive strength, indirect tensile strength, freeze and thaw effect, and impact resistance. Table 3 shows the results of the measured properties.

### 3.1. Workability

**Figure 6:**
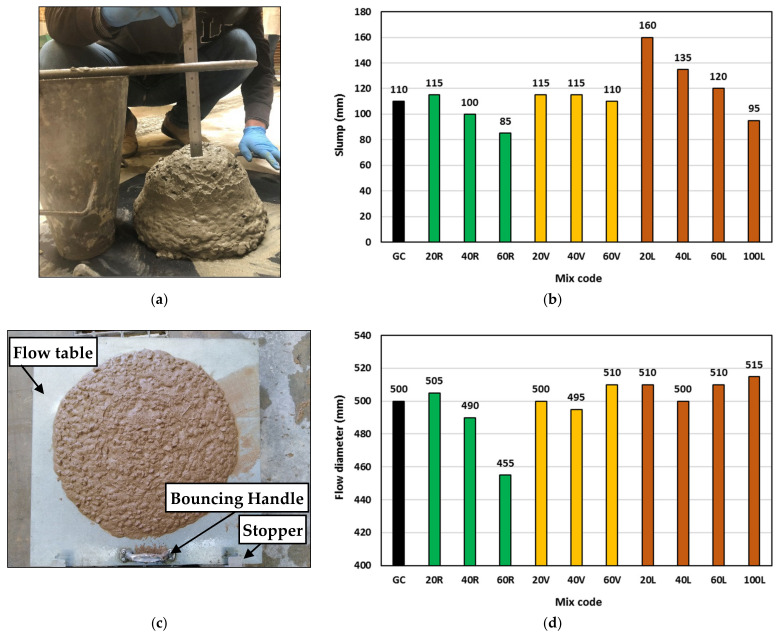
Concrete workability: (**a**) slump measurement, (**b**) slump values, (**c**) flow diameter measurement, and (**d**) flow diameter values.

The concrete workability was measured through the traditional slump test and the flow diameter test according to AS 1012.3.5 [34]. The average flow diameter was determined after filling, lifting the standard slump cone and vibrating the concrete on the flow table by lifting and dropping one side of the flow table (37 mm travel) 15 times. Figure 6 shows the details and results of measured concrete workability. Generally, replacing concrete sand by lightweight materials showed a clear effect on the concrete slump, but not on the concrete flowability under vibration. Compared with the control GC concrete, using 20% rubber (mix 20R), slightly increased the geopolymer concrete slump by only 4%; however, as the rubber content increased further (mixes 40R and 60R), the slump started to decrease by 9% and 23% at 40% and 60% rubber content, respectively. The interesting phenomenon of slump increase then decrease might be attributed to the relatively low ability of rubber to absorb water and its hydrophobic nature that could increase the free water content within the concrete matrix at low rubber content. However, at higher rubber content, the particle texture and shape effect was more pronounced. Compared with that of sand, the poorly shaped rubber particles with the relative light weight of the overall mix led to slower movement within the concrete matrix and hence slump reduction [35]. This was not the case when using vermiculite, where the geopolymer concrete showed up to 5% increase in the slump regardless of the vermiculite content. The insignificant effect of the vermiculite on geopolymer concrete slump might be attributed to its relatively very light weight and highly compressible nature compared with sand, which could allow other heavy materials (like dolomite stone) in the mix to compress/crush its particles and reduce its size within the concrete matrix and hence marginalize its effect. The LECA performed in a similar way to rubber, where the slump increased at 20% LECA and then started to decrease with LECA content increase. Compared with the control GC mix, replacing 20%, 40%, and 60% of GC sand by LECA, increased the concrete slump by 45%, 22%, and 9%, respectively. Complete replacement of sand by LECA in mix 100 L decreased the concrete slump by 14%. The fineness modulus of the LECA particles used was 5.3 compared with 2.2 for the replaced sand. This meant relatively larger particle size and smaller surface area of LECA compared with sand which might increase the free water content within the concrete matrix and hence, the slump increase. However, with the LECA content increase, the possibility of absorbing a higher amount of water increased and therefore, higher slump losses occurred.

The flow table test results did not show clear differences between the tested mixes such as those observed from the slump results. All mixes showed an average flow diameter of around 500 mm, as shown in Figure 6d. This indicated the ability of the geopolymer concrete mixes to flow well under vibration. The flow diameter of the rubber mixes (20 R, 40 R, and 60 R) showed a similar trend to their slump, where the flow decreased with increasing rubber content, but still within 50 mm (10%) difference. Both vermiculite and LECA mixes showed almost constant flow diameter values regardless of the lightweight material content, with 10 mm (2%) difference when using vermiculite (mixes 20 V, 40 V, and 60 V) and 15 mm (3%) difference when using LECA (mixes 20 L, 40 L, 60 L, and 100 L).

### 3.2. Unit Weight

**Figure 7:**
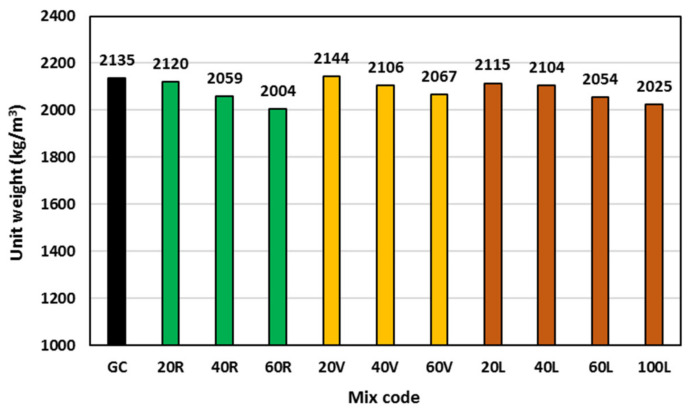
Unit weight of all geopolymer concrete mixes.

Figure 7 shows the measured average unit weight for all geopolymer concrete mixes. As expected, by increasing the lightweight material content in the geopolymer concrete mix, the mix unit weight decreased. Replacing up to 60% of geopolymer concrete sand decreased the concrete unit weight by 6%, 3%, 4% for rubber, vermiculite, and LECA, respectively. Although the vermiculite is the lightest material used in this study, it showed the lowest ability to reduce the geopolymer concrete unit weight. This can be attributed to its highly compressible nature that could allow the dolomite stone to squeeze or crush its particles within the concrete matrix and marginalize its effect [36]. Complete replacement of geopolymer concrete sand by LECA decreased the unit weight by 5% compared to the control GC mix.

### 3.3. Compressive Strength

**Figure 8:**
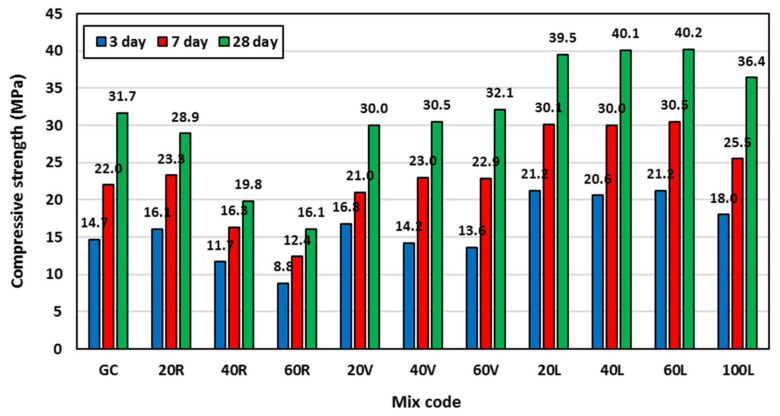
Concrete compressive strength at different concrete ages for all mixes.

The compressive strength was measured in this study at concrete age of 3, 7, and 28 days to compare the development of geopolymer concrete strength with different lightweight fine aggregates. Figure 8 shows all the measured average compressive strengths. At 20% rubber content, the compressive strength was enhanced by 10% and 6% at 3 days and 7 days, respectively compared to the GC mix. However, the strength decreased by 9% at 28 days. The increase of compressive strength at early ages using rubber might be attributed to the relatively low ability of rubber to absorb water and its hydrophobic nature that could increase the free water content within the concrete matrix and provide early self-curing of geopolymer concrete and hence, relatively higher early strength [37]. However, the concrete weakness caused by rubber particles due to the relatively low adhesion at the interface between rubber and the concrete matrix was more pronounced at later concrete age. Replacing 40% and 60% of geopolymer concrete sand by rubber decreased the compressive strength by 20% and 40%, respectively at 3 days, by 26% and 44%, respectively at 7 days, and by 38% and 49%, respectively at 28 days. This was again due to the relatively low adhesion between rubber and the surrounding concrete matrix resulting from its hydrophobicity and its relatively low stiffness, which adversely affected the penetration of the rubber aggregate by the binder paste [38]. This causes the rubber to separate easily from the surrounding concrete matrix under small stresses leading to early cracking and strength reduction.

The measured average compressive strength of geopolymer concrete with vermiculite as a partial replacement of sand showed scattered results regardless of the vermiculite content. At 20% vermiculite content, the compressive strength increased by 14% at 3 days and decreased by 5% at both 7 and 28 days. At 40% vermiculite content, the compressive strength decreased by 3% at 3 days, increased by 5% at 7 days, and decreased by 4% at 28 days. At 60% vermiculite content, the strength decreased by 7% at 3 days, increased by 4% at 7 days, and increased by 1% at 28 days. The scattering of compressive strength using vermiculite might be attributed to the relatively very light weight and highly compressible nature of vermiculite compared with sand. This could allow the dolomite stone as a heavy material in the mix to compress the vermiculite particles and reduce their size, hence creating a degree of instability in the mixtures [36]. Based on these results, it is not recommended to use vermiculite in a cementitious matrix (like cement mortar) with heavy materials (like stone or gravel) in its ingredients.

The performance of geopolymer concrete that utilized LECA as partial or full replacement of sand was different from what was observed when using rubber or vermiculite, in that it showed higher compressive strength than that of the GC mix at all ages and all replacement ratios. The average strength increase ranged between 22% and 44% at 3 days, 16% and 39% at 7 days, and 15% and 27% at 28 days. The higher compressive strength of LECA geopolymer concrete was attributed to the higher fineness modulus of LECA compared with sand. The LECA used had fineness modulus of 4.3 compared with 2.2 for the replaced sand. The higher the aggregate fineness modulus, the higher the compressive strength of concrete [39]. 

The rubber particles used had similar fineness modulus to that of LECA (fineness modulus of rubber was 4.85). However, rubber showed an adverse effect on the 28 day compressive strength. This is mainly attributed to the difference in the rigidity of the aggregate particles. Rubber particles are flexible in nature which results in low adhesion at the rubber/binder interface, and this increases with the increase of rubber content and hence compressive strength decrease. On the other hand, LECA particles are relatively rigid and have porous surfaces, which results in good penetration of their surfaces by the mortar and hence high adhesion at the LECA/binder interface.

### 3.4. Indirect Tensile Strength

**Figure 9:**
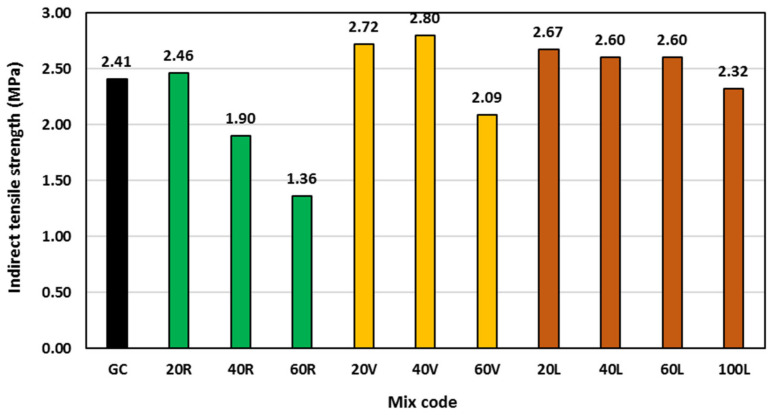
Indirect tensile strength at different concrete ages for all mixes.

The 28 day indirect tensile strength was determined and plotted in Figure 9. Up to 20% rubber content, there was no obvious influence on the tensile strength compared with the corresponding GC mix. Beyond that, the tensile strength started to decrease. Replacing 40% and 60% of geopolymer concrete sand by rubber decreased the indirect tensile strength by 21% and 40%, respectively. At low rubber content, the rubber particles might be able to bridge the cracks under loading which overcame the expected tensile strength reduction corresponding to the reduction in compressive strength. With increasing rubber content, the number of weak points within the concrete matrix became more pronounced due to the poor adhesion between rubber and binder, and hence leading to easier cracking under tensile stresses [38].

The tensile strength of vermiculite mixes correlated to the corresponding 28 day compressive strength, where the tensile strength increased with increasing vermiculite content. All measured indirect tensile strengths of vermiculite mixes were higher than that of the control GC mix by 13%, 16%, and 18% at 20%, 40%, and 60% vermiculite content, respectively. This might be attributed to the deformability of the vermiculite particles that could reduce the crack initiation and propagation in geopolymer concrete under tensile loading and hence lead to higher tensile strength. 

Similar to the compressive strength pattern of LECA geopolymer concrete mixes, their indirect tensile strength increased by a constant value of 8–11% up to 60% LECA content, then started to decrease by 4% at 100% LECA content. The reasons for LECA geopolymer concrete tensile strength increase then decrease were the same as those for the compressive strength, due to the strong correlation between them. 

### 3.5. Freezing and Thawing Effect

**Figure 10:**
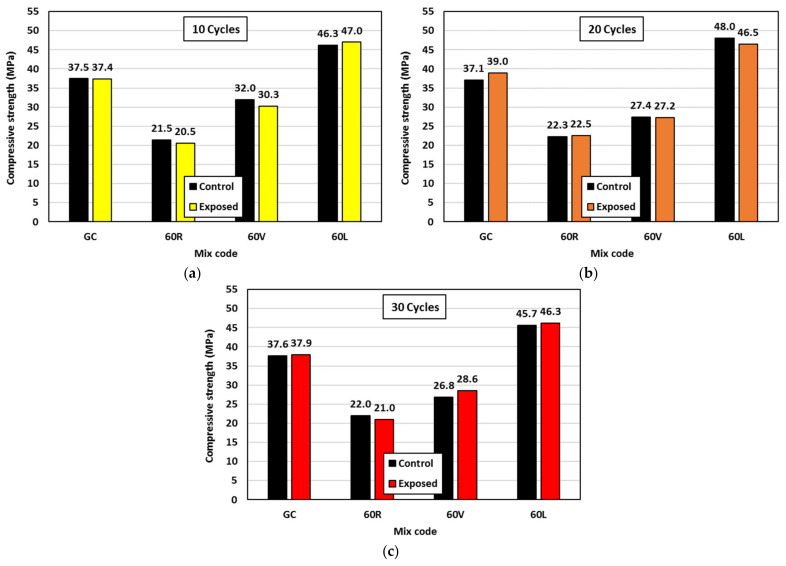
Effect of freezing-thawing cycles on geopolymer concrete 100 mm3 cube compressive strength: (**a**) 10 cycles, (**b**) 20 cycles, and (**c**) 30 cycles.

The durability of geopolymer concrete was measured by exposing 100 mm^3^ concrete cubes to freezing-thawing cycles [30]. This was conducted on mixes GC, 60 R, 60 V, and 60 L. The compressive strength of the exposed cubes to the freezing-thawing cycles (after 10, 20, and 30 cycles) was compared with that of control cubes taken from the same mixes, see Figure 10. As can be observed from the results, there was no significant effect on the compressive strength up to the maximum applied number of freezing-thawing cycles (30 cycles). It is recommended to test geopolymer concrete with lightweight fine aggregate under a higher number of cycles for better investigation of that effect.

### 3.6. Impact Resistance

**Figure 11:**
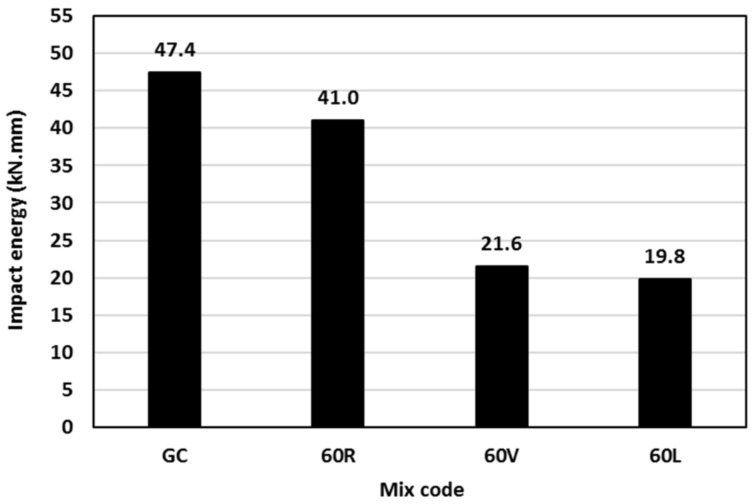
Impact resistance of geopolymer concrete mixes.

The geopolymer concrete impact resistance was measured for mixes GC, 60 R, 60 V, and 60 L through a drop weight impact test using ten discs from each mix [31]. The results of the tested mixes are shown in Figure 11. Regardless of the lightweight fine aggregate type used, the impact resistance of geopolymer concrete mixes decreased when replacing 60% of the sand volume. The geopolymer concrete with rubber showed only 13% decrease in the impact resistance although its corresponding compressive strength decreased by 50%, as shown in Figure 8. This is unlike the rubber performance in Portland cement concrete in which the impact resistance increases with the rubber content increase [40]. The geopolymer concrete with vermiculite or LECA behaved similarly in that they showed about 57% decrease in the impact resistance compared with that of mix GC, although their compressive strengths were even higher than that of the control mix GC. The results indicated that the rubber was able to show sustainable impact resistance when used in geopolymer concrete compared with other lightweight fine materials. This could be due to the superior toughness and ductility of rubber compared with other materials used, which could work as springs within the concrete matrix [41]. This helped to absorb energy and dissipate it rather than transfer to the surrounding materials and therefore, postpone the initiation of the concrete cracks.

### 3.7. Reinforced Slabs Behavior 

#### 3.7.1. General Observations and Failure Mode

**Figure 12:**
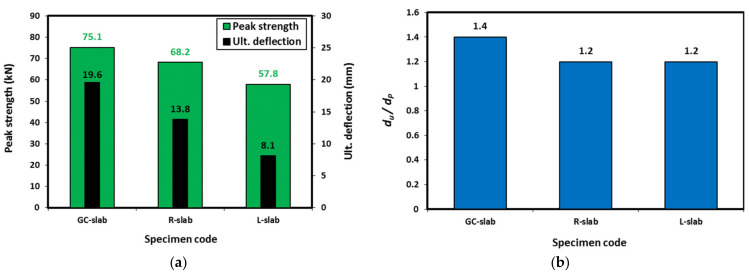
Results of the tested slabs: (**a**) peak strength and ultimate deflection, and (**b**) displacement ratio.

The results of tested one-way slabs under 4-point bending are presented in Table 4. The ultimate displacement in this table is defined as the measured displacement when a drop of 20% occurred in the strength after reaching its peak strength. Figure 12a shows the ultimate displacement and the peak strength of the tested slabs. Although these slabs were made of concrete with similar axial compressive strengths, using lightweight fine aggregates significantly affected the flexural performance of the tested slabs. Compared with the GC-slab, using rubber and LECA decreased the slab strength capacity by 9% and 23% and the deflection capacity by 30% and 59%, respectively. This might be attributed to less adhesion of rubber/LECA with geopolymer material compared with sand, which may lead to easy separation of those lightweight materials under the direct tension caused by the bending stresses [12].

Figure 12b and Table 4 show the ratio of the ultimate displacement (d_u_) to the displacement at peak strength (d_p_). Compared with the GC-slab, both the R-slab and the L-slab showed 14% lower d_u_/d_p_ ratio as the d_u_/d_p_ ratio decreased from 1.4 to 1.2. This generally indicates the lower ductility of lightweight geopolymer concrete in this study compared with the normal weight geopolymer concrete at a given compressive strength.

**Figure 13:**
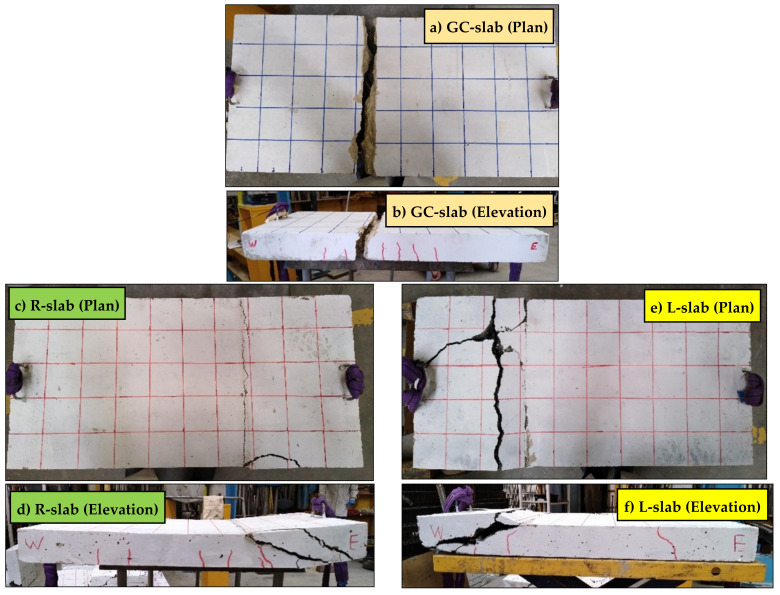
Global damage, crack extension, and failure mode of the tested slab specimens: CC-slab in (**a**,**b**), R-slab in (**c**,**d**), and L-slab in (**e**,**f**).

Figure 13 shows the global damage, crack extension, and failure mode of the tested slab specimens. For the GC-slab, at about 20 kN load (corresponding to an applied bending moment of 3.3 kNm), flexural cracks initiated at the tension zone within the area located between 300 mm from west side and 400 mm from east side. With continued loading, the cracks extended through the full slab depth and a complete rupture of the steel reinforcement occurred at about 420 mm from the west side. Relatively fewer bending cracks were observed in both the R-slab and L-slab, as their failure mode was due to a concrete shear failure. Shear cracks initiated at both east and west sides of each slab. This occurred at load of 11 kN in the R-slab and 14 kN in the L-slab. Some bending cracks were observed in the R-slab within 100 mm east of the slab mid-span. The main shear crack that caused slab failure extended between the support and the loading point from the slab east side in the R-slab and from the slab west side in the L-slab. Therefore, it can be concluded that partial or full replacement of geopolymer concrete sand by lightweight fine aggregate adversely affected the shear resistance of concrete and can cause early failure of slabs, without benefiting from the full capacity of the bending reinforcement.

#### 3.7.2. Load-Deflection Behavior

**Figure 14:**
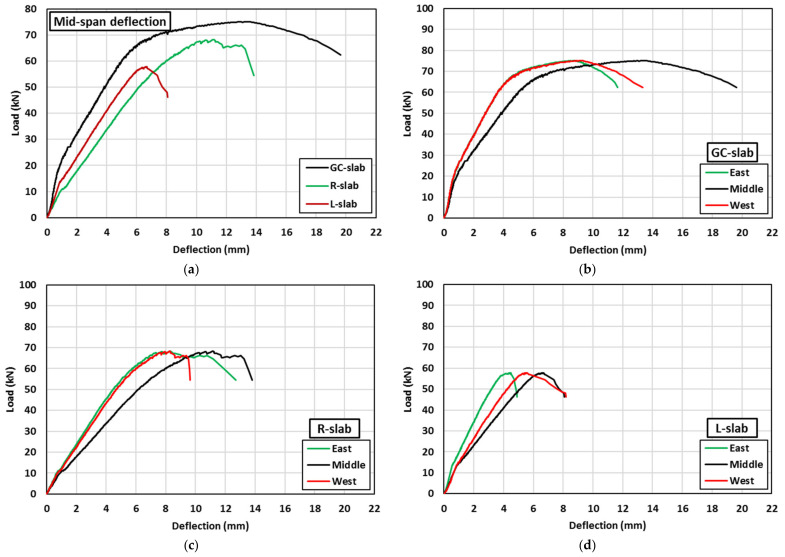
Load-deflection behaviour at several locations for the tested slabs: (**a**) comparison of all slabs, (**b**) GC-slab, (**c**) R-slab, and (**d**) L-slab.

Figure 14 shows the recorded load versus slab deflection at different locations for tested slabs. All slabs showed bilinear behavior connected by a small transition zone. The transition behavior was followed by nonlinear behavior until the end of the test. The transition zone was attributed to the initiation of the cracks in the concrete tension zone that led to a reduction in slab stiffness. The nonlinear behavior was due to the cracking of concrete, crack width increase, spalling of concrete cover, and/or yielding of longitudinal bars. GC-slab specimens were able to resist the load until initial cracks occurred in the concrete tension zone at about 20 kN load and 0.9 mm mid-span deflection, see Figure 14a. This was followed by a reduction in slab stiffness and then the slab was able to resist more load (the load kept increasing) until the slab mid-span deflection approached about 5 mm at a load of 62 kN, where the steel reinforcement started to yield. This caused a rapid increase in the mid-span deflection with slow increase in the load until approaching peak load of 75.1 kN at deflection of 13.5 mm. Then, slab stiffness degradation with load decreasing commenced until a complete rupture of steel reinforcement occurred at the slab’s mid-span, causing failure at 62.2 kN and 19.6 mm.

The stiffness of both the R-slab and L-slab were less than the GC-slab throughout the test. This was due to the fact of easy cracking of geopolymer concrete containing lightweight materials. The R-slab showed less stiffness than the L-slab due to the flexibility that rubber contributed to concrete when partially replacing natural sand. In R-slab and L-slab specimens, the slabs were able to resist the load until initial cracks occurred at about 11 kN load and 1.2 mm mid-span deflection for R-slab and 14 kN load and 1.0 mm mid-span deflection for L-slab. This was followed by a reduction in slab stiffness and then the load kept increasing until the slab mid-span deflection approached 6.5 mm at a load of 52 kN for R-slab and the mid-span deflection approached 5.6 mm at a load of 54 kN for L-slab. At this stage, clear shear cracks started to appear and became significant at the east side of R-slab when the slab reached its peak load of 68.2 kN at mid-span deflection of 11.1 mm. Similarly, the shear cracks became significant at the west side of L-slab when the slab reached its peak load of 57.8 kN at mid-span deflection of 6.6 mm. Once the R-slab and L-slab reached their peak loads, rapid increase was observed in the east deflection of R-slab, see Figure 14c, and in the west deflection of L-slab, see Figure 14d, with slab stiffness degradation and load decreasing. The stiffness degradation in the R-slab was smoother than that in the L-slab. The R-slab failed in shear at a load of 54.5 kN and east deflection of 12.7 mm. The L-slab failed in shear at a load of 46.2 kN and west deflection of 8.2 mm. From the above observations, it can be concluded that partial or full replacement of geopolymer concrete sand by lightweight fine aggregate decreases the slab stiffness and increases the rate of post-peak load degradation.

#### 3.7.3. Toughness

**Figure 15:**
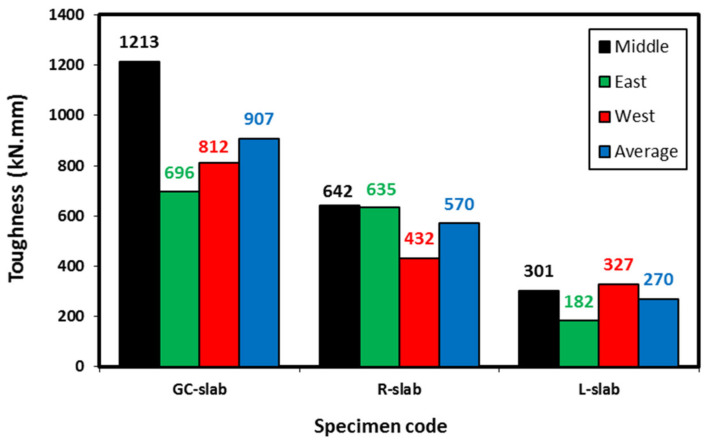
Toughness of the tested specimens.

The area under the load-deflection curves of each slab specimen has been determined to represent the toughness, shown in Table 4 and plotted in Figure 15. The toughness was determined using the deflections measured at the middle, east, and west of each slab, to represent the effect of slab deformations on toughness at different locations. The average deflection was also used to determine the toughness to compare with that determined at the slab mid-span. For the GC-slab specimen, the measured toughness at slab mid-span was higher than those measured at the slab’s east and west sides. This reflects the failure mode, which was bending failure close to the mid-span of the slab where the largest deflection was recorded and therefore the largest measured toughness. As the failure of the GC-slab occurred close to the slab mid-span from the west side, as shown in Figure 13, the slab showed higher toughness at the west side than at the east side. The calculated average toughness of GC-slab was about 76% of its mid-span toughness. 

The R-slab displayed east toughness close to its mid-span toughness. The L-slab performed similarly but for its west toughness. This was again related to the location of failure of each slab in which the slabs started to deflect more at their middles, but once the load approached their shear capacities, the deflection increased at one of the slab sides and hence reflected on the measured toughness. Replacing 20% of geopolymer concrete sand decreased its middle, east, and west toughness by 47%, 9%, and 47%, respectively. Full replacement of geopolymer concrete sand by LECA decreased its middle, east, and west toughness by 75%, 74%, and 60%, respectively. Although both types of sand replacements showed less toughness than the corresponding GC-slab, the R-slab exhibited better toughness than the L-slab. This is attributed to the flexibility rubber can add to the geopolymer concrete, which increased its strain capacities and hence led to better toughness. The calculated average toughness of both R-slab and L-slab were about 89% of their mid-span toughness, which is higher than that shown by the GC-slab. This was due to the relatively higher deflections that occurred at east or west locations of those slabs (R-slab and L-slab) compared with their middles, hence higher average toughness.

From the results above, it can be concluded that the performance of lightweight geopolymer concrete in toughness is lower than the normal weight geopolymer concrete having similar compressive strength. Some additive to the lightweight geopolymer concrete to enhance its flexural performance might be recommended for future research and comparisons.

#### 3.7.4. Slab Deflection Pattern

**Figure 16:**
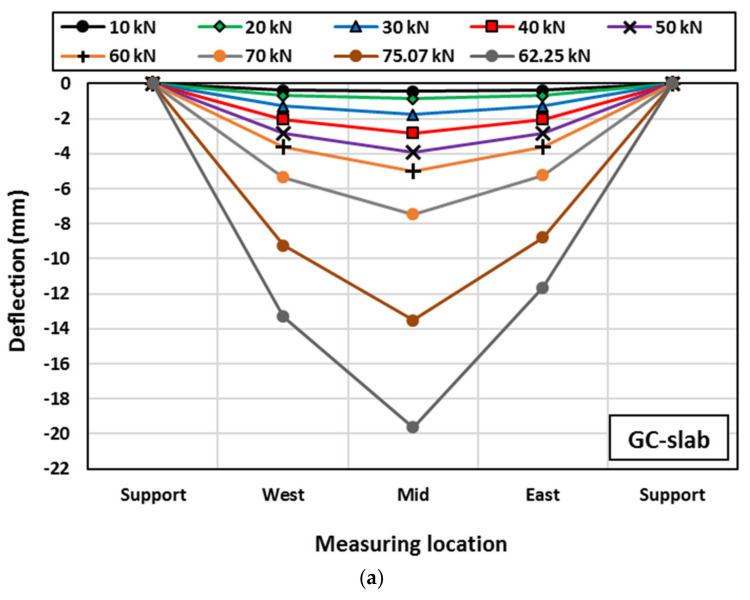

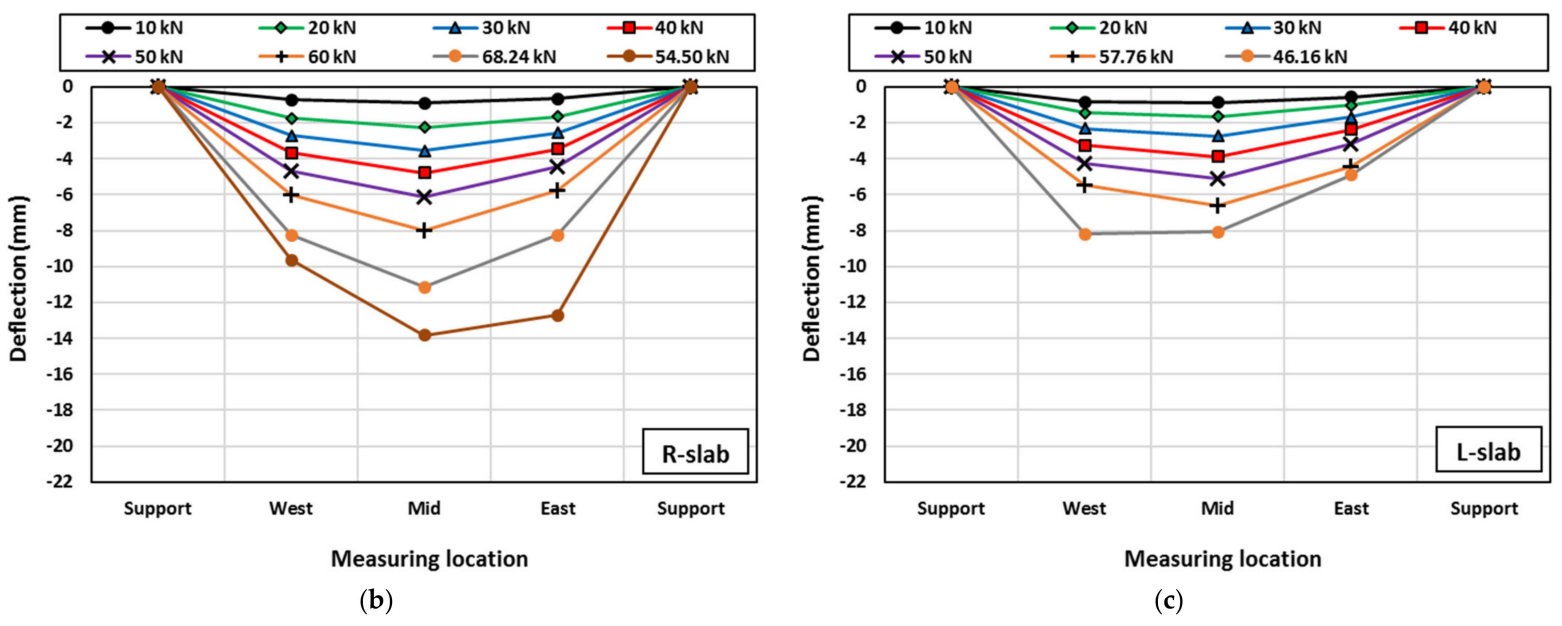
Deflection distribution at each 10 kN for: (**a**) GC-slab, (**b**) R-slab, and (**c**) L-slab.

Three LVDTs were used to measure the deflection of each slab at locations named East, Middle, and West as shown in Figure 5. The measured deflection distribution for each 10 kN up to the peak load and at the failure load is shown in Figure 16. As can be observed, with the applied load increase, the slab deflections increased with symmetrical distribution along the slab span up to loads of 70 kN, 68.24 kN, and 40 kN, respectively for GC-slab, R-slab, and L-slab. Beyond those loads, the failure cracks started to initiate, and hence unsymmetrical deflection was observed. Specimens GC-slab and L-slab showed deflections at the west side higher than those at the east side, and vice versa for specimen R-slab. The location of higher deflection had no relationship with the concrete type, but it was more related to initiation and development of the cracks until failure.

**Figure 17:**
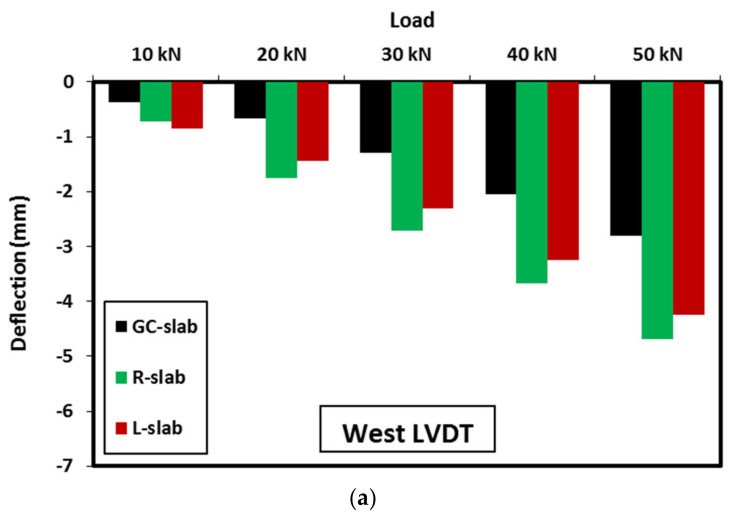

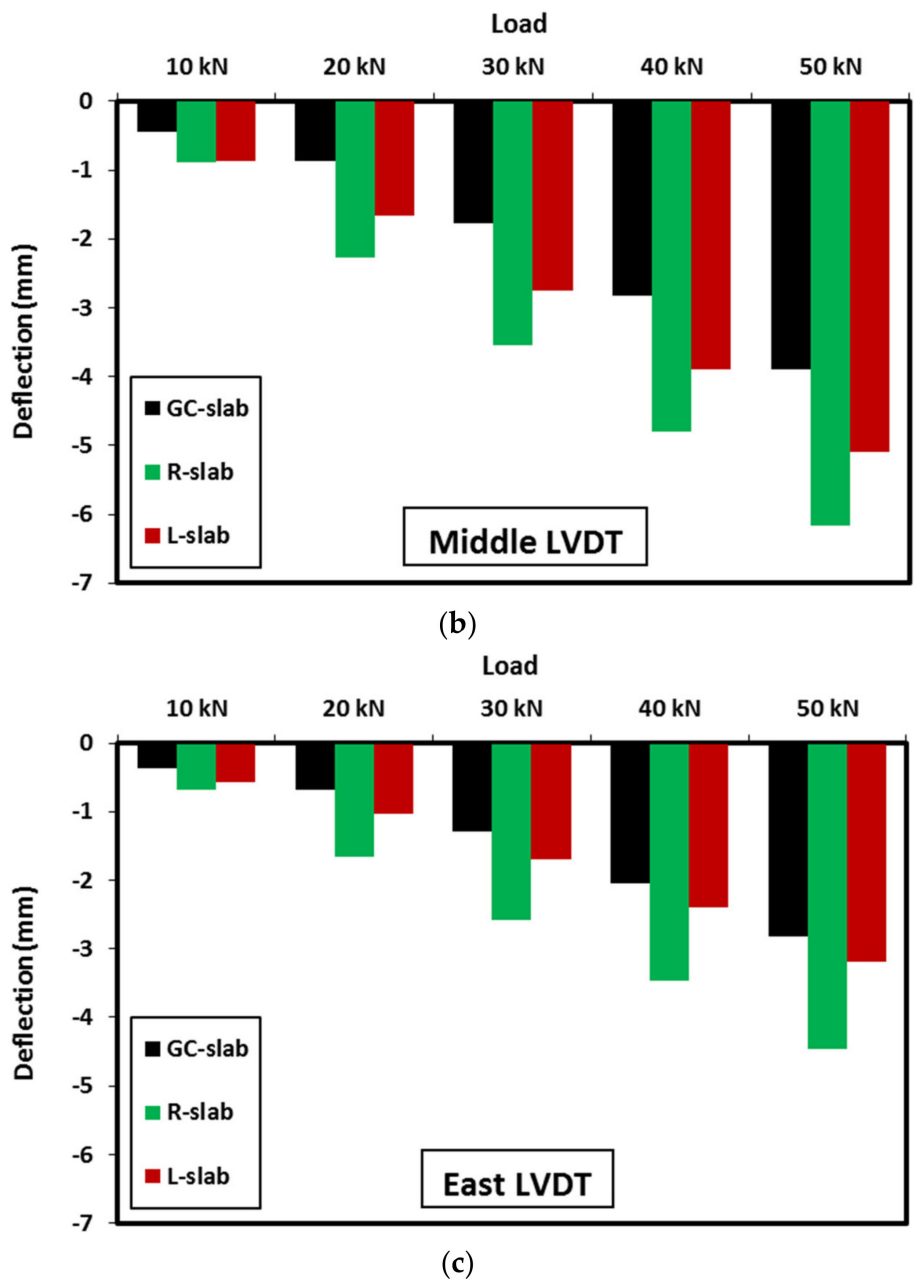
Comparison of slabs deflection at given loads: (**a**) West, (**b**) Middle, and (**c**) East.

Figure 17 shows a comparison of the slab deflections at given loads up to 50 kN, as this was the mutual range among all slabs. As shown, at any given load and any location, the R-slab and L-slab were able to deform more than the GC-slab. This might be attributed to the deformability of the lightweight materials used compared with the replaced sand. At almost all given loads and any location, the R-slab displayed clearly higher deflection than that shown by the L-slab, especially at middle and east locations. This is attributed to the high flexibility of rubber particles which increases the slab deformability under loads. This might be a useful feature for geopolymer concrete if used in structures resisting seismic loads that need deformable concrete rather than high strength concrete. From this discussion, it can be concluded that in regard to the deflection of the slab, using lightweight materials in geopolymer concrete causes higher deflection under flexural loading.

## 4. Conclusions and Recommendations

This research investigated the mechanical performance, durability and structural behavior of geopolymer concrete (GC) containing lightweight materials. Eleven GC mixes were produced, tested, compared, and analyzed. The variables in this study included the lightweight fine aggregate material (rubber, vermiculite, and LECA), and the GC sand replacement ratio (20%, 40%, 60%, and 100%) by volume. The measured fresh and hardened concrete properties and durability characteristics were workability, compressive strength at several ages, tensile strength, freeze and thaw effect, and impact resistance. The structural behavior of GC was also investigated in reinforced slabs made of selected mixes and subjected to flexural loads. The key findings of this study can be summarized as follows:

i. Replacing concrete sand by lightweight materials showed a clear effect on the GC slump, but not on its flowability under vibration. This indicated the ability of the lightweight GC mixes to flow well under vibration.
ii. GC compressive strength increased when using 20% rubber content but decreased beyond that. Regardless of the vermiculite content, it showed scattered effect on the GC compressive strength. LECA showed significant increase in GC compressive strength by up to 44%, 39%, and 27% at 3, 7, and 28 days, respectively.
iii. There was no significant effect on the GC compressive strength when applying freezing-thawing cycles (up to 30 cycles) in this study. Regardless of the lightweight fine aggregate used, the impact resistance of GC mixes decreased when replacing 60% of the sand volume.
iv. The slab strength and deflection capacities decreased by 9% and 30%, respectively when using rubber, and by 23% and 59%, respectively when using LECA, compared with the control GC slab
v. Partial or full replacement of GC sand by lightweight fine aggregate adversely affected the shear resistance of concrete and caused early shear failure of slabs. In addition, it decreased the slab stiffness and increased the rate of post-peak load degradation.

Of the tested mixes, GC mix with 60% LECA (mix 60L) showed better mechanical performance than that shown by other mixes in this study. This indicates the applicability of this type of lightweight GC in structures subjected to axial loads. However, rubber would be the best lightweight material to recommend in this study to partially replace GC sand to resist impact and flexural loads. It is recommended for future studies to test the proposed GC mixes for durability, including drying shrinkage, water absorption, surface abrasion, carbonation, and conducting microstructural analyses.

## Figures and Tables

**Table 1:** Chemical composition of geopolymer binders.

Geopolymer Binder	CaO (%)	SiO_2_ (%)	Al_2_O_3_ (%)	Fe_2_O_3_ (%)	SO_3_ (%)	MgO (%)	Na_2_O (%)	K_2_O (%)	SrO (%)	TiO_2_ (%)	P_2_O_5_ (%)	Mn_2_O_3_ (%)	LOI (%)
Fly ash	5.8	51.1	18.1	9.7	1.0	7.3	3.9	1.8	0.1	0.8	0.2	<0.1	0.2
Slag	43.1	32.8	13.4	0.4	1.9	5.5	0.4	0.3	0.8	0.6	<0.1	0.1	0.8

**Table 2:** Ingredients of concrete mixes (kg/m^3^).

Mix	Fly Ash	Slag	Cement	Activator	Water	Dolomite	Sand	Rubber	Vermiculite	LECA	SP	Retarder
GC	260	260	0	207	177	611	611	0	0	0	9.4	2.07
20R	260	260	0	207	177	611	489	45	0	0	9.4	2.07
40R	260	260	0	207	177	611	367	91	0	0	9.4	2.07
60R	260	260	0	207	177	611	245	136	0	0	9.4	2.07
20V	260	260	0	207	177	611	489	0	11	0	9.4	2.07
40V	260	260	0	207	177	611	367	0	22	0	9.4	2.07
60V	260	260	0	207	177	611	245	0	34	0	9.4	2.07
20L	260	260	0	207	177	611	489	0	0	79	9.4	2.07
40L	260	260	0	207	177	611	367	0	0	159	9.4	2.07
60L	260	260	0	207	177	611	245	0	0	238	9.4	2.07
100L	260	260	0	207	177	611	0	0	0	397	9.4	2.07

**Table 3:** Experimental results.

Mix Code	Slump (mm)	Flow Diameter (mm)	Unit Weight (kg/m^3^)	Compressive Strength (MPa)			Indirect Tensile Strength (MPa)	Freezing-Thawing Effect (MPa)						Impact Resistance	
								10 Cycles		20 Cycles		30 Cycles		Mean (Blows)	Energy (kN.mm)
				3D	7D	28D	28D	C	E	C	E	C	E		
GC	110	500	2135	14.7	22.0	31.7	2.41	37.5	37.4	37.1	39.0	37.6	37.9	10.8	47.4
20R	115	505	2120	16.1	23.3	28.9	2.46	--	--	--	--	--	--	--	--
40R	100	490	2059	11.7	16.3	19.8	1.90	--	--	--	--	--	--	--	--
60R	85	455	2004	8.8	12.4	16.1	1.36	21.5	20.5	22.3	22.5	22.0	21.0	9.3	41.0
20V	115	500	2144	16.8	21.0	30.0	2.72	--	--	--	--	--	--	--	--
40V	115	495	2106	14.2	23.0	30.5	2.80	--	--	--	--	--	--	--	--
60V	110	510	2067	13.6	22.9	32.1	2.09	32.0	30.3	27.4	27.2	26.8	28.6	4.9	21.6
20L	160	510	2115	21.2	30.1	39.5	2.67	--	--	--	--	--	--	--	--
40L	135	500	2104	20.6	30.0	40.1	2.60	--	--	--	--	--	--	--	--
60L	120	510	2054	21.2	30.5	40.2	2.60	46.3	47.0	48.0	46.5	45.7	46.3	4.5	19.8
100L	95	515	2025	18.0	25.5	36.4	2.32	--	--	--	--	--	--	--	--

C: Compressive strength of Control specimen; E: Compressive strength of Exposed specimen.

**Table 4:** Reinforced geopolymer concrete slabs test results.

Specimen Code	Mix Code	Compressive Strength (MPa)	Peak Strength (kN)	Def. at Peak Strength, dp (mm)	Ult. def., du (mm)	(du/dp)	Toughness (kN.mm)			
							Middle	East	West	Average
GC-slab	GC	31.7	75.1	13.5	19.6	1.4	1213	696	812	907
R-slab	20R	28.9	68.2	11.1	13.8	1.2	642	635	432	570
L-slab	100L	36.4	57.8	6.6	8.1	1.2	301	182	327	270

## Data Availability

Data is contained within the article.
